# ProxiMAX randomization: a new technology for non-degenerate saturation mutagenesis of contiguous codons

**DOI:** 10.1042/BST20130123

**Published:** 2013-09-23

**Authors:** Mohammed Ashraf, Laura Frigotto, Matthew E. Smith, Seema Patel, Marcus D. Hughes, Andrew J. Poole, Husam R.M. Hebaishi, Christopher G. Ullman, Anna V. Hine

**Affiliations:** *School of Life and Health Sciences, Aston University, Aston Triangle, Birmingham B4 7ET, U.K.; †Isogenica Ltd, The Mansion, Chesterford Research Park, Little Chesterford, Essex CB10 1XL, U.K.

**Keywords:** antibody engineering, gene library, MAX randomization, overlap PCR, protein engineering, saturation mutagenesis, CDR3, complementarity-determining region 3

## Abstract

Back in 2003, we published ‘MAX’ randomization, a process of non-degenerate saturation mutagenesis using exactly 20 codons (one for each amino acid) or else any required subset of those 20 codons. ‘MAX’ randomization saturates codons located in isolated positions within a protein, as might be required in enzyme engineering, or else on one face of an α-helix, as in zinc-finger engineering. Since that time, we have been asked for an equivalent process that can saturate multiple contiguous codons in a non-degenerate manner. We have now developed ‘ProxiMAX’ randomization, which does just that: generating DNA cassettes for saturation mutagenesis without degeneracy or bias. Offering an alternative to trinucleotide phosphoramidite chemistry, ProxiMAX randomization uses nothing more sophisticated than unmodified oligonucleotides and standard molecular biology reagents. Thus it requires no specialized chemistry, reagents or equipment, and simply relies on a process of saturation cycling comprising ligation, amplification and digestion for each cycle. The process can encode both unbiased representation of selected amino acids or else encode them in predefined ratios. Each saturated position can be defined independently of the others. We demonstrate accurate saturation of up to 11 contiguous codons. As such, ProxiMAX randomization is particularly relevant to antibody engineering.

## Background

Saturation mutagenesis (replacement of wild-type codons with codons for all 20 amino acids) is a core technique within the protein engineer's repertoire. Its importance in engineering non-native ligand-binding domains is undisputed. Thus saturation mutagenesis has played a vital role in creating synthetic zinc-finger-based transcription factors [1,2], antibodies and antibody-derived scaffolds [3,4] for many years, and, more recently, has proved valuable in engineering modified enzymes [5].

Conventional saturation mutagenesis employs degenerate codons NNN, NNK or NNS. Although technically undemanding, degeneracy leads to significant problems that have provoked a drive for non-degenerate alternatives. Traditionally, non-degeneracy was achieved by using trinucleotide phosphoramidites which add whole codons (rather than single bases) during oligonucleotide synthesis [6]. In 2003, we described ‘MAX’ randomization [7], which uses standard oligonucleotides and is useful for enzyme engineering, but requires separation between randomized codons and thus cannot saturate more than two contiguous codons. Recently, simpler alternatives of ‘small-intelligent libraries’ designed by the program DC-analyzer [8] and the ‘22c trick’ [9] have been described, which are optimal methodologies to saturate small numbers of codons effectively and efficiently, irrespective of location; although, owing to multiplex PCR primers, these approaches cannot saturate larger numbers of codons (see below). In the present paper, we describe ProxiMAX randomization, which offers all the advantages of Slonomics™ [10,11] (an automated, non-degenerate, enzyme-based process), but can be performed in a standard molecular biology laboratory. ProxiMAX likewise combines the benefits of non-degeneracy with the ability to saturate larger numbers of contiguous codons, a key requirement for antibody engineering.

## Saturation mutagenesis: comparison of techniques

**Figure 1:**
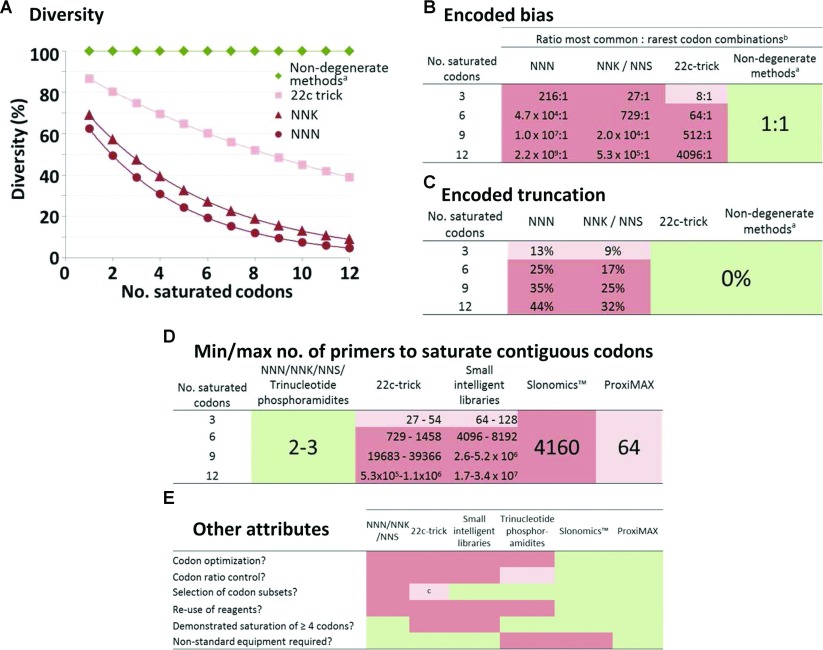
Comparison of performance of common saturation mutagenesis techniques Green coloration indicates ideal performance, pale pink coloration indicates tolerable performance, and deep pink coloration indicates unacceptable performance, where non-degenerate methods (^a^) include ‘small-intelligent libraries’ [8], Slonomics™ [10,11] and ProxiMAX. (**A**) Diversity was calculated using the formula *d*=1/(*N*Σ_k_*p*_k_^2^) [12] and is in agreement for a 12-mer peptide saturated with codon NNN [13]. (**B**) Ratios represent the theoretical relative concentrations of each individual gene combining any of the most common codons (leucine/arginine/serine, NNN/NNK; or leucine/valine, 22c trick) compared with each individual gene containing any combination of the rarest codons (^b^) [methionine/tryptophan, NNN; cysteine/aspartate/glutamate/phenylalanine/histidine/isoleucine/lysine/methionine/asparagine/glutamine/tryptophan/tyrosine, NNK; or 18 codons (omitting leucine/valine), 22c trick]. (**C**) Truncation is calculated as the percentage of sequences that contain one or more termination codons within the saturated region. (**D**) NNN/NNK/trinucleotide oligonucleotides may be used as a DNA cassette, or as primers in PCR-based mutagenesis; Slonomics™ and ProxiMAX require a fixed number of oligonucleotides and the numbers of primers for the 22c trick [9] and small-intelligent libraries [8] were calculated using the formulae in the respective publications for saturating consecutive codons. (**E**) ^c^As dictated by degeneracy limitations.

The advantages and disadvantages of the various approaches to saturation mutagenesis are compared in Figure 1. The most critical consequences of degeneracy are the loss of diversity/functionality [12,13] and inherent encoded bias [7]. Diversity is a measure of the percentage of unique species within a library. Even the ‘22c trick’ [9] (NDT/VHG/TGG degeneracy) leads to >60% loss of diversity over 12 saturated codons (Figure 1A). It might be argued that excess screening capacity (e.g. with ribosome or CIS display [14]) diminishes this issue, but in libraries with more than three randomized positions, the use of degenerate codons will probably have a severe impact on the quality of the output. Since the magnitude of protein–ligand interactions is a function of both affinity and concentration, all display screens are based on a pretext of equivalent concentration of library members. Figure 1(B) demonstrates that the concentration differences between common and rare codon combinations are unworkable beyond three degenerate saturated codons, regardless of methodology. Library diversity is further restricted by NNK and NNN saturations which randomly introduce termination codons (Figure 1C) that may lead to non-functional proteins, possibly leading to aggregation. Practical issues must also influence method choice. Notwithstanding objections of bias, the number of primers required to saturate more than three consecutive codons using either ‘small-intelligent libraries’ or the ‘22c trick’ are impractical to handle manually (Figure 1D). Neither do degenerate methods (including the ‘22c trick’) allow the potential to exclusively eliminate cysteine, which is usually undesired in protein and peptide libraries. Finally, Figure 1(E) compares other desirable attributes of saturation techniques, including the ability to select codons to suit the organism of choice, including codon optimization, ratio-control, subset-selection, etc. Thus we propose that ProxiMAX is the first technology to offer all desirable attributes in a manual setting and, as such, will be an invaluable addition to the protein engineer's toolbox.

## ProxiMAX randomization: the concept

**Figure 2:**
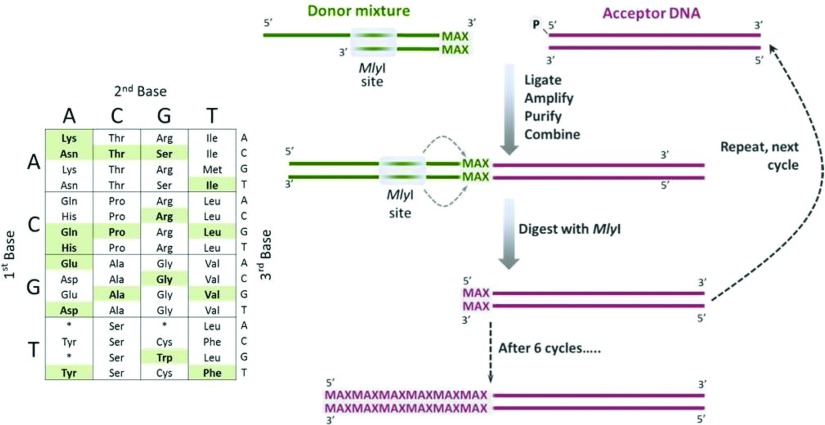
ProxiMAX methodology Double-stranded DNA donors, carrying the required ‘MAX’ codons at their termini, are ligated individually on to a double-stranded DNA acceptor sequence (phosphorylated at the required 5′ end only). The donors can take the form of partially double-stranded DNA (as shown), fully double-stranded DNA or hairpin oligonucleotides. After ligation, the products are amplified, purified, quantified and then combined in the required ratios. The combined product is digested with MlyI. The process is then repeated, using the digestion product from cycle 1 as the acceptor for the next round of ligation. Different sets of donors are cycled to prevent potential carry-over from one cycle to the next. The inset of the genetic code shows that any codons may be selected as ‘MAX’ codons, regardless of their sequence.

ProxiMAX randomization is based on iterative cycles of blunt-ended ligation, amplification and digestion with the Type IIS restriction endonuclease MlyI. More specifically, oligonucleotide donors (containing the required codons at their termini) are ligated on to a conserved acceptor sequence. Following ligation, the PCR amplification step provides ample ligated product, while concomitantly diluting non-ligated contaminants. Subsequent digestion removes all but the final codon of the donor sequences to generate a new acceptor, which enters the next cycle (Figure 2). By alternating different sets of oligonucleotide donors for each cycle, codon additions can be restricted to a specific saturation cycle.

## ProxiMAX development: T4 DNA ligase exhibits some sequence preference in blunt-end ligations

To examine process feasibility, preliminary experiments used a mixture of all 20 donors combined in nominally equal quantities (based solely on manufacturer's data). Results demonstrated that the process worked, but, unsurprisingly, there was bias in the resulting small-scale library (Supplementary Figure S1 at http://www.biochemsoctrans.org/bst/041/bst0411189add.htm). Bias might result from variant oligonucleotide quality, inaccurate estimations of oligonucleotide concentration, sequence preferences of T4 ligase, amplification bias during PCR or even sequence preference of the restriction enzyme MlyI. To examine and/or exclude these factors, ligations and amplifications were performed in individual parallel reactions. Only after purification and quantification were the individual amplicons combined. Analysis at this midpoint in each of six cycles of saturation (i.e. after combination of individual reactions and before MlyI digestion) demonstrated that equimolar mixes (5% of each codon) had been generated successfully during each cycle of addition (Supplementary Figure S2A at http://www.biochemsoctrans.org/bst/041/bst0411189add.htm). However, these data offered no insight as to whether subsequent ligations would proceed in an equimolar fashion when those mixtures acted as acceptors. Therefore, after six cycles of saturation mutagenesis, composition of the final assembled library was assessed by Roche 454 pyrosequencing, which demonstrated that, whereas most codons were introduced into the library at the expected ratio, a few were noticeably over- and under-represented. In particular, the histidine codon 5′-CAT-3′ was favoured, whereas codons for lysine and threonine were under-represented (Supplementary Figure 2B).

By eliminating donor concentration and/or quality and PCR preference as potential sources of bias, these data suggest that T4 DNA ligase exhibits some sequence preference in blunt-ended ligations, when presented with a mixture of 5′ and 3′ ends, so that, in this substrate mixture, the enzyme has a different reactivity rate for different codons. To examine this possibility, data from the preceding 6-mer sequence analysis were studied for expected and observed ratios with respect to the last base in the phosphate donor/acceptor junction. A χ^2^ statistic with one degree of freedom was calculated for the 16 possible combinations of 3′–5′ preference at the ligation junction and where *P*<0.0001, this was noted (Supplementary Table S1 at http://www.biochemsoctrans.org/bst/041/bst0411189add.htm). Although there were no discernible rules for the junction preference directly at the point of ligation, there are certainly preferences for 3′-T and 5′-C, which supports the observed over-representation for histidine (CAT), but does not fully explain the reason for histidine being more preferred than arginine (CGT).

## Successful application of ProxiMAX randomization

**Figure 3:**
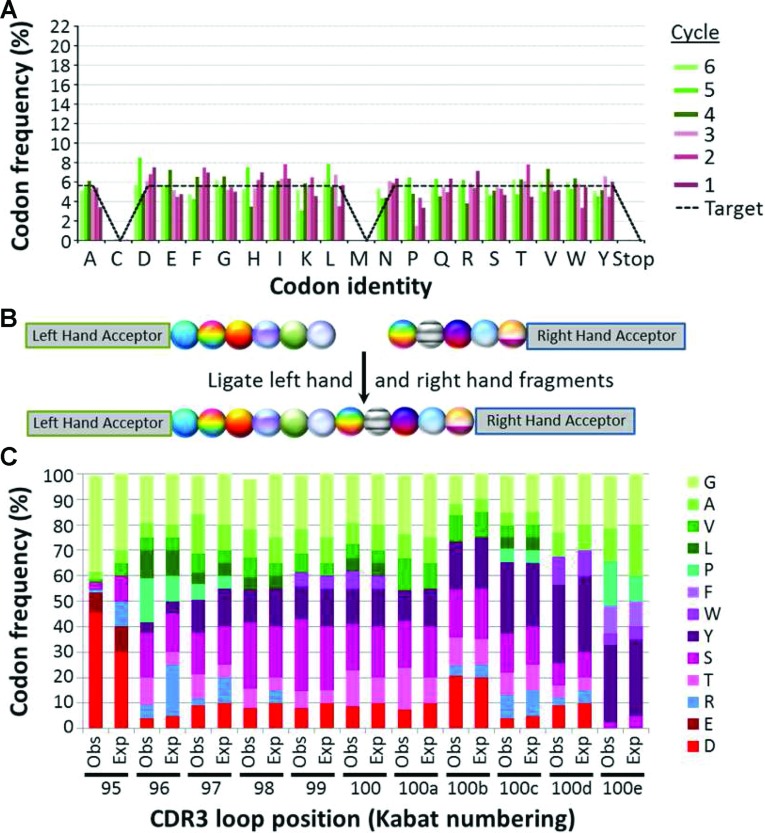
Library construction with controlled codon ratios (**A**) DNA sequence analysis of a library encoded by six cycles of codon addition, optimized to provide unbiased representation. Six cycles of ProxiMAX randomization were undertaken as described in Supplementary Figure S2 (at http://www.biochemsoctrans.org/bst/041/bst0411189add.htm), using right-handed hairpins (Supplementary Table S3 at http://www.biochemsoctrans.org/bst/041/bst0411189add.htm) as donors and an amplicon of pUC19 as the acceptor, with optimized mixtures of 18 codons adjusted to reflect the sequence bias illustrated in Supplementary Figure S2(B). The resulting library was analysed by DNA sequencing, using a MiSeq DNA sequencer according to the manufacturer's instructions. Data represent the analysis of 286684 sequences of the correct length, which represented 79.9% of the entire library. Bars represent the frequency of each codon from each cycle of saturation mutagenesis. The broken line depicts the target representation for each codon. Further analysis of the library can be found in Supplementary Table S2 (at http://www.biochemsoctrans.org/bst/041/bst0411189add.htm). (**B**) Schematic representation of the procedure for assembling a representative 11-mer CDR3 antibody library. Left-hand (LH, six cycles) and right-hand (RH, five cycles) of codon additions were performed in parallel as described in Supplementary Figure S2(B) with adjusted ratios of codons to compensate for sequence preference as determined in Supplementary Figure S2(B). After the final MlyI digestions, the two assemblies were joined together to generate a region of 11 contiguous saturated codons. (**C**) DNA sequence analysis of the representative 11-mer CDR3 antibody library. The library was assembled as described in (**B**) and sequenced using a MiSeq sequencer according to the manufacturer's instructions. Data represent the analysis of 461274 sequences of the correct length, which comprised 74.1% of the entire library. Single codon deletions comprised an additional 7.4% of that library, whereas double and triple codon deletions comprised 2.0% and 1.2% respectively. Expected (designed) frequencies of each codon are compared with observed frequencies for locations 95–100e within the CDR3 loop. Further analysis of the library can be found in Supplementary Table S2.

To compensate for these apparent sequence preferences, the six cycles of addition were repeated, with the concentration of each donor sequence being adjusted during mixing, to counteract the over- or under-representation shown in Supplementary Figure S2(B). Thus concentrations of donors carrying over-represented codons were reduced, whereas those carrying under-represented codons were increased. Cysteine and methionine codons were also excluded as these are deemed to have liabilities in peptide libraries by being susceptible to oxidation. It is clear from Figure 3(A) that T4 DNA ligase sequence preference can be compensated for by adjustment of donor concentrations.

Having demonstrated successful equimolar representation, we next sought to create a more relevant library from a protein engineering perspective. Saturation of 11 consecutive codons within the loop of a CDR3 (complementarity-determining region 3) domain of an antibody V_H_ (variable heavy) chain domain was undertaken. Each position in the loop required a separate mixture of codons so that favourable protein-binding amino acids were included at the correct positions and those that would encode structural or manufacturing liabilities were excluded. The frequencies of the desired amino acids were rounded to the nearest 5% in order to simplify mixing of the codon oligonucleotides and analysis of the final library.

For this longer loop, both right-hand and left-hand acceptors were employed to build two library components in parallel (Figure 3B). The 11-mer CDR3 loop was then assembled by ligating the two fragments. The loop was sequenced, and remarkable compliance was achieved between design specifications (expected) and the actual library generated (observed; Figure 3C). One notable anomaly was the arginine codon which was poorly represented in the left-hand acceptor ligations. This is apparent in Figure 2(C), where the lack of under-representation of arginine at a particular addition has led to a compensating over-representation of other amino acids, for example aspartate at position 95. Despite this, the majority of codons are remarkably close to their desired frequency.

## Conclusions

Although more involved than conventional degenerate saturation mutagenesis, we believe that the benefits of ProxiMAX randomization easily outweigh the increased outlay on reagents and of time. One to two cycles of ProxiMAX randomization can be achieved in a day and thus our exemplar 11-mer library can readily be prepared in 2 weeks. The bottleneck of directed evolution is library screening [9] and thus the smaller and more accurate the initial library, the greater the efficiency of sampling in selection or screening and the better the likely outcome. As stated by Neugebauer and co-workers (Morphosys AG) “The most critical parameter for all *in vitro*-based approaches is the quality of the antibody library” [15]. Thus although the requirement to perform individual ligations and PCRs may initially seem unwieldy, performing 20 parallel reactions from a single mastermix is not a significant undertaking. The phrase ‘if you want a good result, preparation is nine-tenths of the work’ was never more apposite.
